# Criminalised, victimised or other? A reflexive engagement with Queer Criminology utilising a relational pedagogical approach

**DOI:** 10.3389/fsoc.2024.1373422

**Published:** 2024-04-22

**Authors:** Liam Wrigley, Evangelia Koumentaki

**Affiliations:** ^1^School of Criminology, Investigation and Policing, Leeds Trinity University, Leeds, United Kingdom; ^2^School of Social Sciences, Keele University, Keele, United Kingdom

**Keywords:** Queer Criminology, flipped classroom, LGBTQIA+, SOTL, Higher Education

## Abstract

Queer Criminology is a newfound area of exploration within the discipline of Criminology, which is uniquely positioned to deal with issues regarding crime and victimisation concerning those from the LGBTQIA+ community and gender diverse/minoritized groups. The field of “Queer Criminology” has become vast and expanding, having explored issues of interpersonal, structural and systematic inequality concerning those from the community and beyond. To this end, narratives of victimisation, trauma and injustice have dominated (and limited) understandings of Queer Criminology. Moreover, limited thinking has been attributed within the Scholarship of Teaching and Learning (SOTL), which seeks to understand LGBTQIA+ individuals and groups—beyond binarized thinking of victimhood or criminalised. In this article, we offer the perspectives of two higher education professionals teaching Queer Criminology in a “flipped” classroom environment, which positions the learner as expert within the subject matter and utilises a relational pedagogy lens to do so. We discuss the use of our reflexive practice, as both Feminist Decolonial and Queer Criminologists. The article touches upon trauma informed approaches to teaching Queer Criminology. We offer several steps in building a coalition of learning, which can unpick the potential policy, theory, and practical tensions of teaching Queer Criminological Scholarship.

## Introduction

Criminological scholarship has been broadly situated across the domains of Criminal Justice, Social-Legal Studies, Psychology and Sociology related to the study of offenders, victims and the study of Crime. It is important to understand, however, that the Criminological omissions or ignorance of particular social groups is not new (Woods, 2014; Ball, 2016). Indeed, feminist scholarship has pointed towards the dominance of sexism and patriarchy in criminology and, therefore, the neglect of gendered experiences and knowledge production (Smart, 1977; Carlen, 2017). Due to androcentrism and heteronormativity shaping Criminology, the interest in LGBTQIA+ (*Lesbian, Gay, Bisexual, Trans, Queer, Intersex and Asexual*) narratives has been largely absent from the discipline—until recently (Morris, 2018; Rogers and Rogers, 2023). As Woods (2014, p. 16) notes “*the bulk of LGBTQ-inclusive criminological research from the past four decades has focused almost exclusively on bias crime/bullying and intimate partner violence*”. Our intention in this article is not, however, to juxtapose such path breaking scholarship. Instead, it is our intention, to expand the “*Criminological Imagination*” (Young, 2011) and detail our perspectives as educators within Criminology and the Applied Social Sciences, where we can contribute towards better understanding Queer and LGBTQIA informed Criminological narratives, in coalition with classroom-based learners.

Firstly, we engage with an opening discussion of Queer Criminology. Secondly, the article engages with our positionality as Queer and Intersectional feminist educators in Criminology, and how we reflexively engage with learners in fostering inclusivity and equality in teaching Criminology. Thirdly, we engage with the SOTL practice literature and highlight the relative knowledge gaps in relation to teaching Queer Criminology as a trauma informed and flipped and relational pedagogical practice (Bovill, 2020). Finally, we give our perspectives from a relational pedagogical queer standpoint and how we have worked towards building a coalition of learning within classroom and SOTL practices.

## Criminalised, victimised or other? Queering criminology

As briefly mentioned, for several scholars, criminological concerns have focused on “sexual deviance” and patterns of violence that have privileged heteronormativity via epistemological orientations of investigation, which focus predominantly on heterosexuality and heteronormative assumptions (Ball, 2014, 2016; Dwyer et al., 2016; Rogers and Rogers, 2023). Indeed, since the 1970s, criminology, and latterly victimology, has provided a plethora of evidence from distinguished scholars, which have predominantly focused on criminogenic understandings of the family, women-centred experiences of crime and male-pattern violence within the domestic sphere (see Stanley and Wise, 1979; Carlen, 2017; Cook and Walklate, 2019; Rutter and Barr, 2021). Several of these pioneering branches within Criminology have informed policy-based evidence making, police recorded crime statistics, self-disclosed victimisation surveys, international and national crime surveys (Cunneen, 2023). Some of which, have thus far overlooked non-heteronormative patterns of criminality, victimisation, and victimhood (Ball, 2016; Colliver and Silvestri, 2022). Allied scholarship from the branches of feminist and intersectional criminology have attempted to alleviate some of these concerns through centering cross-cutting issues crucial to criminology and the study of victims, which includes inequalities delineated across race/ethnicity, gender, and sexuality (Yuval-Davis, 2006; Crenshaw, 2013). More so, Intersectional Criminology has highlighted the propensity for Criminal Justice Systems in the Global North to reproduce the racism and gender-based discrimination within its interactions with criminalised individuals and victims of crime, that Criminology has attempted to explore and eliminate (Potter, 2013).

Nonetheless, the absence and omission of Queer Criminological thinking within a UK Higher Education context remains stark. As such, there remains a lack of focus on the lives of LGBT and Queer people in relation to crime and victimhood (Ball, 2014, 2016). In England and Wales alone, hate related crimes against Gay and Lesbian people in 2021/22 jumped to 25,639 from 14,161 in 2018/19 and almost doubled for the Trans community from 2,253 in 2018/19 when compared to 4,262 reported incidents in 2021/22 (Home Office, 2023). We also acknowledge the under-reporting and (lack of) confidence in criminal justice agencies, in relation to the reporting of crimes against the LGBTQIA+ community and issues with administrative data in explaining the complexities of such reports (see also Zempi et al., 2021). Therefore, we position students as experts in their own learning and life experiences.

Given some of these issues, however, it is essential to recognise and voice LGBTQIA+ victimisation within Criminological knowledge production. Consequently, as Woods (2014, p. 16) acknowledges, ‘*there is a need for criminologists to investigate the diversity of circumstances under which LGBTQ people experience and commit crime'*. It is our perspective in this article, however, that we need to go much further in understanding how Queer Criminological thinking is produced in the classroom and explored with learners. Through unpacking and cementing their knowledge acquisition regarding Queer issues, in the safety of a classroom environment, we position learners as co-investigators and experts in developing critical pedagogy within the flipped classroom environment that centres Queer knowledge production (Malcom and Lloyd, 2024). To do this, we utilise the lens of relational pedagogy, which builds upon the relationship between learner, peers and lecturer for effective learning. As Bovill (2020, p. 3) points out “(relational pedagogy) puts relationships at the heart of teaching and emphasises that a meaningful connection needs to be established between teacher and students as well as between students and their peers”.

## A note on reflexivity within queering the classroom: our positionality

Reflexivity is an important tool that is embedded throughout learning and teaching practices (Cain et al., 2022). Reflexivity encourages dialogue that considers beliefs, interests, experiences and values that may impact upon the classroom environment. Willig (2001, p. 1) advises that “personal reflexivity” guides us to be critical about given assumptions and beliefs. The tacit knowledge that comes from being personally reflexive helps to shape our interactions with classroom-based learners. In doing so, this has helped both educators and learners acknowledge how our own subjectivities and lived experiences influence SOTL practices in and outside the classroom environment (Archer, 2009). As such, we both acknowledge and have embedded reflexivity in their approach to teaching Queer Criminological issues. In “Queering” the classroom, we give our reflexive perspectives as Higher Education professionals, who have experienced trauma related to hate crime, homophobia, violence, racism, and xenophobia throughout our time in and outside of academic settings.

For instance, Liam Wrigley (he/him) is a working-class Queer Criminologist and Applied Social Policy academic with an expertise in researching young people's lives. He has worked as Researcher and Lecturer across several institutions in the UK. Liam Wrigley successfully completed his doctoral studies in Sociology and Social Policy at the University of Sheffield in 2022 (see Liam Wrigley, 2024). Before entering academia, he worked within several statutory youth support settings and as a volunteer and researcher, including a LGBTQIA+ organisation that has supported young people and their families (including families of choice) in navigating homelessness, homophobia, transphobia and hate crime. Liam Wrigley is a survivor of youth violence and has experienced disablist micro-aggressions, ableism, institutional homophobia, victim-blaming, and negative assumptions associated with the intersections of his working-class identity. He has experiences of police-initiated encounters, as a victim of violent crime and as a professional working in Higher Education.

On another note, Evangelia Koumentaki (she/her) is an early-career Critical Criminologist with expertise in indigenous/customary forms of justice and punishment. After working in the private sector and offering her support to young victims, Evangelia Koumentaki left her country to pursue her dream of obtaining postgraduate studies in Criminology. She successfully completed her doctoral studies in Criminology at the University of Essex in 2022. Prior to her doctoral studies, Evangelia Koumentaki coordinated research projects on Restorative Justice and other topics related to criminology. Evangelia Koumentaki experienced racism during the early years of her life and throughout her educational, personal, and professional life due to her cultural heritage. Sexism, discrimination, and harassment in her life also have occurred as a “courtesy” of her gender. Having experienced such forms of hate, Evangelia Koumentaki is committed to fighting any form of discrimination and oppression, aiming for inclusiveness, and contributing to social justice through her teaching and research.

## Background: teaching queer criminology as a flipped classroom SOTL practice

During academic year 2022–23, Liam Wrigley and Evangelia Koumentaki led a series of interactive lectures to MA level degree students within a medium sized UK Higher Education Institution. Both Liam Wrigley and Evangelia Koumentaki were responsible for several undergraduate and postgraduate teaching provisions within Criminology. Both authors have since taught Queer Criminology several times since, in a number of different institutions. Here, we reflect on our working relationship, where we explored the postgraduate taught and undergraduate provision and identified several gaps in areas of Criminological scholarship that had not been delivered within such programmes. With students being shortly introduced to feminist criminological scholarship in the early months of their studies, further teaching and familiarisation with gender related subjects were substantially reduced. We were both able to identify that some modules and teaching opportunities lacked an understanding of Queer, Intersectional, and wider Equality, Diversity, and Inclusion issues.

As Feminist and Queer Critical Criminologists, we both operated a deep ethic of care towards our colleagues and students across all our working relationships (see also Stanley and Wise, 1979). As reflexive practitioners, we drafted several learning and teaching resources that were attuned to our learners' voices/needs and recognised professional and interdisciplinary boundaries between learner, peer group and teaching staff as a form of good relational pedagogy (Bovill, 2020). It is our perspective throughout that our professional relationships are key to unpacking and understanding discursive issues of queerness, trauma and feelings of difference in the safe space of a classroom learning environment. However, we quickly come to understand that we should not impose a framework of learning upon students, and instead, should be able to learn in a de-hierarchical environment (Cornelius-White and Harbaugh, 2009). Our perspective is that the student should be experts of their own lived experiences that enrich teaching and learning opportunities, therefore we decided to “flip” the classroom and support students in developing their own critical disciplinary literacy around understandings of Queer Criminology (see also Ahearne, 2022).

We understand that students may not have conscious experiences with the LGBTQIA+ community or may possess unconscious biases of such. We therefore asked that students build a series of rules of engagement that promotes safety, respect, understanding and inclusion when engaging with each other's perspectives. Through building a relational pedagogical approach throughout the academic year, we quickly came to know students who had experienced trauma because of LGBTQIA+ victimisation. In part, this was achieved through both Liam Wrigley and Evangelia Koumentaki being open about our experiences of victimisation and encouraging dialogue around our interpersonal knowledge of such within the professional boundaries of the virtual and physical classroom.

It is important to note that there is no prior demographic data within student systems that captures protected characteristics such as Lesbian, Gay, Bisexual, Trans or Queer identity in England and Wales. However, notable exceptions include the possibility of disclosure if a student chooses to be in learner inclusion plans, whereby students are known to student services (Cain et al., 2022). This raises further complexities as sexuality and gender identity are sometimes disaggregated from student demographic profiles that increases the possibility of Higher Education professionals working within assumptions and bias. For instance, a student disclosing being a victim of a Homophobic or Transphobic hate crime (see also Rogers and Rogers, 2023)—this does not give an accurate understanding of a student's LGBTQIA+ identity or provide Higher Education professionals prior knowledge of students who have experienced trauma, victimhood or criminality related to gendered identity or sexuality. In many ways, this could promote the potential for bias or assumption within professional practice. To also note, “*sexual orientation*” and discrimination in relation to one's “*sex*” is a protected characteristic under the Equality Act (2010) Section 12, which we understand requires a more nuanced analysis which is beyond the scope of this article. Both authors reflect upon flipped classroom activities in a way which makes use of advanced content warnings and reminders to engage with care, reflection and understanding of each student's needs. We also worked in a way which did not assume identity or protected characteristics and upheld our position as responsible and ethical educators. We build upon trauma informed practices and good relational practices between learner groups.

## Flipping the classroom: lessons from queer relational pedagogy in action

Importantly, Queer criminology has arrived to counteract heteronormative modes of knowledge production in criminological thinking and, consequently, to open a window of inclusivity for queering victim and offender dichotomies (see Ahmed, 2006; Rogers and Rogers, 2023). We, however, argue that such different voices and experiences, nonetheless, are hard to understand when Higher Education institutions do not have an appropriate grounding in working with Queer related issues, or a solid procedural understanding of the complex intersectional inequalities that LGBTQIA+ learners face. As maintained throughout this perspective article, it is our reflection that learning is an evolving process and has been positioned best when we are relatable to learners and colleagues and can foster inclusivity in our working relationships. Here, we reflect on our perspectives as Higher Education professionals teaching Queer Criminological issues and steps taken to create a relational pedagogical approach.

## Active understanding of institutional dynamics in relation to queer criminology

One step towards challenging heteronormative processes within Criminology programmes is to actively support LGBTQIA+ students in their progression through visible ally ship. We note that throughout our careers, we have actively championed the voices of LGBTQIA+ students alongside other key intersectional dimensions of difference, such as gender and ethnicity/race. We understand that in more cases than often, undergraduate programmes recruit high numbers of White cisgender female students, with such accomplishments actively celebrated throughout our careers inter alia institutional widening participation agendas (see also Trebilcock and Griffiths, 2022). As a cisgender de-colonial feminist scholar, Evangelia Koumentaki has critically questioned such administrative endorsements by pointing out that although the institution has promoted inclusive strategies to welcome female students, queer identities are nonetheless silent within recruitment strategies and final numbers (Colliver, 2021). Evangelia Koumentaki argues that if we aim for equality, diversity, and inclusion, then we should start counting the numbers of newcomers differently and resist working in silos with colleagues across the institution. Collectively, we position that greater understanding of LGBTQIA+ students' presence and involvement in curriculum design should be acknowledged before entering our classes, to establish an inclusive environment (Henry et al., 2023). By doing this, we do not only promote active inclusive education but more importantly promote democratisation of recruitment practices that consequently will contribute to democratic knowledge processes.

## Working with and unpacking trauma, criminalization, and victimisation from a place of safety

In all our teaching and learning commitments, we have both embedded safety as a paramount approach in tackling Criminological subject issues that come with unpacking trauma. As Higher Education professionals teaching a degree programme, where learners often come to “make sense” of their lived experiences of criminalization and victimisation—Queer Criminology is no different (cf. Dwyer et al., 2016). As this perspectives article has positioned, students come across learning information whereby LGBTQIA+ victims (and perpetrators) voices have been marginalised from mainstream Criminological thinking, which in some cases silences issues related to homophobic and transphobic inequalities within the Criminal Justice system (cf. Ball, 2016). In the dynamics of the classroom environment, we both understand that a large majority of students have engaged with Queer Criminology from a point of care and awareness of the inequalities and othering of the LGBTQIA+ community (Glazzard and Vicars, 2022). In unpacking traumatic experiences, criminalization and victimisation, we position that the classroom should be a safe environment to build relational pedagogical practices that are embedded in promoting trust and meaningful engagement with the learners. By democratising the learning resources through “flipping” the learning content, this gave students the opportunity to share their experiences and build coalitions with other learners in deciphering Queer issues whilst making sense of the subject matter from both a relational and academic perspective.

## Democratising knowledge production and student voice

Finally, we position that knowledge production should be democratised in learning about issues related to Queer Criminology. Democratisation was key in creating a Queer relational pedagogy in the classroom environment. In our teaching we decided to “flip” the classroom environment by giving choice to students regarding the creation of reading lists and democratisation of academic texts, as a way of building relationships with learners. Liam Wrigley notes how he has experienced several difficulties in sourcing appropriate reading list materials, in which Queer Criminological scholarship has not been appropriately financially supported by institutions. Evangelia Koumentaki, on several occasions had to conduct specific orders to institutions to boost reading options on Queer criminology for students who were conducting dissertation projects on related subjects. Therefore, through flipping the learning resources, we allowed students to appropriately express themselves vis-à-vis the reading list creation—such as utilising alternative formats and creative methods, including podcasts, visual resources (such as zines) and vlogs of Queer scholarship. This approach allowed for newer and less established voices to be platformed. We collectively understand that student voice should be promoted by respecting trauma narratives and lived experiences (see also Trebilcock and Weston, 2019; Ahearne, 2021). We also note that the assessment content should be related to Queer offenders and victims by including assessment questions or case studies of Queer criminology relevance. Nonetheless, Queer Criminological thinking should go beyond these binaries and help students to decipher Queer epistemologies, methodologies, and methods to build appropriate disciplinary literacy. As Higher Education professionals, we have made learning materials openly available, instead of gatekeeping the release of information each week via a Virtual Learning Environment (see also Ahearne, 2022). Indeed, democratising knowledge production is not a matter of simply expanding the curriculum but, instead, including queer research and case studies as foundational knowledge early on in an undergraduate degree programme. Going forward, we have decided to introduce Queer subject matters much earlier on in the learning process, through embedding Queer Criminology as first year undergraduate studies; rather than positioning Queer Criminology as a specialist area of interest, or “othering” the subject (Ball, 2016; Vicars, 2020).

## Concluding point

To summarize, both authors have given their perspective points on teaching Queer criminology as a relational pedagogical practice. We have touched upon issues related to trauma, victimisation and criminalization and attempted to recognise and address inequities in learning related to the LGBTQIA+ community in relation to criminology. By utilising a relational pedagogical approach, this has enabled both Liam Wrigley and Evangelia Koumentaki to be reflexive of our praxis as decolonial and queer educators teaching Queer related subject matters. It is important, however, to acknowledge that it is beyond the scope of this perspective article to discuss student-led narratives in understanding the impact of Queer Criminological teaching. We have, nonetheless, harnessed our knowledge of several institutions and the strengths of relational approaches and flipped classroom practices within our teaching. Through providing a pedagogical reflection, we have contributed towards expanding the “Criminological Imagination” towards a Queer centred discipline, which democratically understands student voice and works within safe parameters in unpacking Queer trauma narratives.

## Data availability statement

The original contributions presented in the study are included in the article/supplementary material, further inquiries can be directed to the corresponding author.

## Author contributions

LW: Writing – review & editing, Writing – original draft. EK: Writing – review & editing, Writing – original draft.
